# Protective effect of breastfeeding with regard to children’s behavioral and cognitive problems

**DOI:** 10.1186/1475-2891-13-111

**Published:** 2014-11-29

**Authors:** Subin Park, Bung-Nyun Kim, Jae-Won Kim, Min-Sup Shin, Hee Jeong Yoo, Soo-Churl Cho

**Affiliations:** Department of Psychiatry, Seoul National Hospital, 398Neungdong-ro, Gwangin-gu, Seoul, Korea; Department of Psychiatry and Behavioral Science, Seoul National University College of Medicine, 101 Daehak-No, Chongno-Gu, Seoul, Korea

**Keywords:** Breastfeeding, Attention-deficit hyperactivity disorder, Behavior, Child

## Abstract

**Background:**

Breastfeeding has been associated with a lower risk for behavioral problems in childhood. However, it is uncertain whether these associations are mediated by the mother’s or child’s IQ. We examined the association between breastfeeding and attention-deficit hyperactivity disorder (ADHD) and other behavioral problems in childhood and assessed the role of the child’s IQ and the mother’s IQ in generating this association.

**Findings:**

The current study included 874 children (8-11 years) recruited from schools in five Korean cities. Mothers were asked about nursing, and the prevalence of attention-deficit hyperactivity disorder (ADHD) and behavioral problems were compared between children who were breastfed and those who were not breastfed. After adjusting for age, gender, area of residence, and yearly family income, a lack of breastfeeding was associated with increased internalizing, externalizing, and overall behavioral problems as well as the diagnosis of ADHD. These associations weakened but mostly remained significant after adjusting for child’s IQ and maternal IQ. In addition, a lack of breastfeeding was associated with low child’s IQ and this association weakened, but remained significant even after adjusting for maternal IQ and the diagnosis of ADHD.

**Conclusions:**

This study suggests that there is a protective effect of breastfeeding on childhood behavioral outcomes with a partial mediation of this effect by the child’s IQ, and there is a positive effect of breastfeeding on childhood intelligence with a partial mediation of this effect by the child’s attention problem.

## Background

Studies suggest that there is an association between breastfeeding and a wide range of positive health outcomes in children, including a lower risk of acute ear infections, respiratory tract infections, asthma, obesity, diabetes mellitus, and leukemia [1]. In addition, breastfeeding has been associated with greater cognitive development in childhood [2–6]. In the Port Pirie Cohort Study, Wigg et al. [7] reported that breastfed children had a 1.2 (-2.0 to 4.4) and 0.8 (-1.9 to 3.5) point advantage on the Wechsler Full-Scale IQ test at 7 years of age and 11 to 13 years of age. Breastfeeding has also been associated with attention-deficit hyperactivity disorder (ADHD) [8, 9] and other externalizing behavioral problems [10] and internalizing behavioral problems [10, 11]. However, due to these behavioral outcomes being strongly related to the child’s IQ [12, 13], which is highly associated with breastfeeding [4, 7], it is uncertain whether the association between breastfeeding and the child’s behavioral development is mediated or confounded by the child’s IQ. By contrast, it is possible that inattention problem of children reduce IQ scores by interfering with the child’s capacity to learn in school. For example, Friedman et al. [14] investigated the relation between attention problem at several time points during childhood and IQ scores at age 16 in a large sample, and found longitudinal correlations between -0.21 and -0.27. Similarly, Polderman et al. [15] reported longitudinal negative correlations between attention problem measured by teachers and parents at age 5, and IQ scores at age 12 (r = -0.30 and -0.31, respectively).

In addition, due to the mother’s IQ being highly predictive of both breastfeeding status and the child’s IQ, the mother’s IQ should be considered when examining the association between breastfeeding, the child’s IQ, and the child’s behavioral outcomes [4, 16]. For example, in the 1979 US national longitudinal survey of youth, Der et al. [4] reported that before adjustment for maternal intelligence, breastfeeding was associated with an increase of around 4 points in the child’s IQ, but adjustment for mother's IQ reduced this advantage by 71% to 75%. The results of meta-analysis of eight studies that controlled for maternal IQ also shows that maternal IQ explains most of the effect of breastfeeding, but not all [4].

In this investigation, we attempted to further clarify the complex relationships between breastfeeding, behavioral problems, and the mother’s and child’s IQs among school-aged children in Korea. We tested the following hypotheses: 1) breastfeeding is independently associated with behavioral problems and ADHD, even after controlling for child’s IQ and maternal IQ and 2) breastfeeding is independently associated with child’s IQ, even after controlling for the diagnosis of ADHD and maternal IQ.

## Methods

### Participants and procedure

Participants were recruited from five different administrative regions of Korea: Seoul, Seongnam, Incheon, Ulsan, and Yeoncheon. We selected two to three schools from each region that best represented the local demographics, for a total of thirteen schools, and sent letters to the parents of third and fourth grade children (age range 8-11) inviting them to participate in our study. We gave the parents and children detailed information about the study and then obtained written informed consent from the parents and children. Mothers were asked about feeding methods (i.e. breast, bottle-fed or mixed) in infancy. The child who was breast or mixed feeder was placed into breastfeeding group, and the child who was bottle feeder was placed into non-breastfeeding group. The study protocol was approved by the institutional review board of the Seoul National University Hospital.

We assessed the presence of ADHD in children using a highly structured diagnostic interview, the Diagnostic Interview Schedule for Children Version IV (DISC-IV) ADHD diagnostic module [17]. The DISC-IV was administered to the mothers.We used the Korean version of the Child Behavior Checklist (CBCL) [18, 19] to evaluate behavioral symptoms of children. The CBCL is a parent-report questionnaire by which the child is rated on various behavioral and emotional problems. The CBCL produces three broadband scores (i.e., internalizing, externalizing, and total behavioral problem scores) that can be compared to norms and clinical cutoffs for groups based on age and sex. We considered T-scores of 63 and above to signify clinically significant symptoms based on previous validation studies [19].

The children were individually administered the abbreviated form of the Korean Educational Development Institute’s Wechsler Intelligence Scales for Children (KEDI-WISC) [20]. Each mother completed the short form of the Korean Wechsler Adult Intelligence Scale (K-WAIS) [21].

### Statistical analysis

Group differences in socio-demographic variables between children who were breastfed and children who were not breastfed were evaluated using independent t-tests for continuous variables and chi-squared tests for categorical variables.

To investigate the effect of the child’s IQ on behavioral outcomes, we compared the prevalence of ADHD and the internalizing, externalizing, or overall behavioral problems among children with IQs <100, 100-115, and >115.

To elucidate the association between breastfeeding and the child’s behavioral outcomes, logistic regression tests were performed using the presence of ADHD and either internalizing, externalizing, or overall behavioral problems as the outcome variable and a history of breastfeeding as the principal predictor, after adjusting for gender, age, residential area, and yearly family income (Model 1). Then, models were adjusted for the child’s IQ (Model 2). Finally, the adjustment for maternal IQ was conducted (Model 3). All predictive variables were concurrently entered into the model, and adjusted odd ratio (AOR) for breastfeeding was calculated.

To elucidate the association between breastfeeding and the child’s IQ, a multiple linear regression test was performed using the child’s IQ as the outcome variable and a history of breastfeeding as the principal predictor, after adjusting for gender, age, residential area, and yearly family income. Then, this model was adjusted for maternal IQ. Finally, the adjustment for the diagnosis of ADHD in addition to maternal IQ and other covariates was conducted. Categorical variable was incorporated in the linear regression analyses through dummy coding (e.g., breastfeeding = 0, non-breastfeeding = 1; absence of ADHD = 0, presence of ADHD = 1).

All statistical analyses were performed using SPSS (version 21.0; SPSS Inc., Chicago, IL), and statistical significance was defined at the alpha level <0.05.

## Results

Of the initially recruited 1,089 children, 215 study subjects were excluded due to their responses being incomplete, leaving a total of 874 subjects (80.3%; 509 boys, 365 girls; mean age 9.05 ± 0.71 years) in the analysis. Among these 874 participants, 522 (59.7%) children were breastfed and 352 (40.3%) were not breastfed during infancy. There were no significant differences in socio-demographic characteristics between breastfeeding and non-breastfeeding groups. However, both the mother’s and the child’s IQs were significantly higher in the breastfeeding group than the non-breastfeeding group (Table 1).

**Table 1:** **Socio-demographic characteristics of children who were breastfed and children who were not**

	Breastfeeding (N = 522)	Non-breastfeeding (N = 352)	t or ***X*** ^***2***^	p
Age, mean (SD)	9.07 (0.70)	9.02 (0.72)	0.99	0.323
Male, n (%)	312 (59.8)	197 (56.0)	1.27	0.260
Region			4.56	0.102
Urban	222 (42.5)	129 (36.6)		
Industrial	218 (41.8)	151 (42.9)		
Rural	82 (15.7)	72 (20.5)		
Yearly family income			1.81	0.179
>$25,000	315 (60.9)	229 (65.4)		
<$25,000	202 (39.1)	121 (34.6)		0.683
Maternal IQ	108.11 (11.38)	106.38 (11.89)	2.10	0.036
Child’s IQ	112.41 (13.95)	108.47 (14.16)	4.07	< 0.001

**Figure 1:**
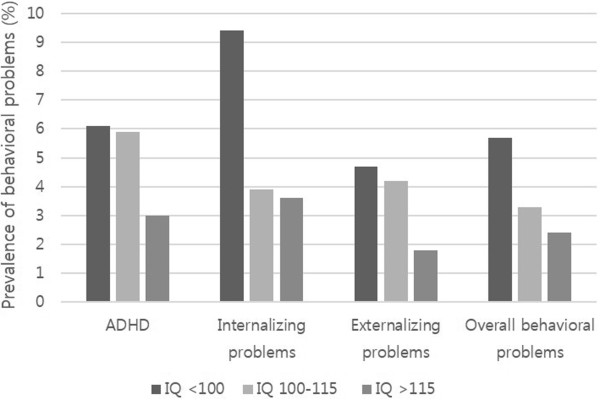
**Prevalence estimates (%) of ADHD and behavioral problems according to the child’s IQ.**

Figure 1 shows the prevalence estimates of ADHD and the morbidity of internalizing, externalizing, or overall behavioral problems according to the child’s IQ. All behavioral problems were the most common in children with IQ <100 and the least common in children with IQ >115, although only the prevalence of internalizing problems was significantly different between IQ groups (X^2^ = 9.33, p = 0.009).

After adjusting for gender, age, residential area, and yearly family income (Model 1), a lack of breastfeeding was associated with increased risk of morbidity of internalizing (AOR = 2.45, 95% CIs = 1.30-4.60), externalizing (AOR = 2.20, 95% CIs = 1.03-4.70) and overall (AOR = 3.36, 95% CIs = 1.53-7.34) behavioral problems as well as the diagnosis of ADHD (AOR = 2.01, 95% CIs = 1.07-3.79). After adjusting for the child’s IQ (Model 2), associations between a lack of breastfeeding and the morbidities of internalizing or overall behavioral problems weakened but remained significant (AOR = 2.30, 95% CIs = 1.22-4.31and AOR = 2.88, 95% CIs = 1.38-6.62, respectively). Significant associations between a lack of breastfeeding and the morbidities of externalizing problems or ADHD were eliminated in this model. These trends preserved even after adjusting for maternal IQ in addition to the child’s IQ and other covariates (Model 3) (Table 2).

**Table 2:** **Associations between breastfeeding and psychiatric and behavioral outcomes**

	Breastfeeding (N = 522)	Non-breastfeeding (N = 352)	Model 1		Model 2		Model 3	
	N, %	N, %	AOR ^a^ (95 % CI)	p	AOR ^b^ (95 % CI)	p	AOR ^c^ (95 % CI)	p
ADHD	19 (3.7)	23 (6.6)	2.01 (1.07-3.79)	0.031	1.81 (0.95-3.43)	0.072	1.78 (0.93-3.41)	0.083
Internalizing problems	17 (3.3)	26 (7.5)	2.45 (1.30-4.60)	0.005	2.30 (1.22- 4.31)	0.010	2.31 (1.22-4.37)	0.010
Externalizing problems	12 (2.3)	17 (4.9)	2.20 (1.03-4.70)	0.042	1.82 (0.86-3.85)	0.117	1.96 (0.87-4.41)	0.105
Overall behavioral problems	10 (1.9)	20 (5.8)	3.36 (1.53-7.34)	0.002	3.02 (1.38-6.62)	0.006	2.87 (1.28-6.42)	0.010

^a^Adjusted for age, gender, area of residence, and yearly family income.

^b^Adjusted for age, gender, area of residence, yearly family income, and child’s IQ.

^c^Adjusted for age, gender, area of residence, yearly family income, child’s IQ, and maternal IQ.

Logistic regression analyses revealed that after adjusting for gender, age, residential area, and yearly family income (Model 1), a lack of breastfeeding was associated with increased risk of morbidity of internalizing (AOR = 2.45, 95% CIs = 1.30-4.60), externalizing (AOR = 2.20, 95% CIs = 1.03-4.70) and overall (AOR = 3.36, 95% CIs = 1.53-7.34) behavioral problems as well as the diagnosis of ADHD (AOR = 2.01, 95% CIs = 1.07-3.79). After adjusting for the child’s IQ (Model 2), associations between a lack of breastfeeding and the morbidities of internalizing or overall behavioral problems weakened but remained significant (AOR = 2.30, 95% CIs = 1.22-4.31and AOR = 2.88, 95% CIs = 1.38-6.62, respectively). Significant associations between a lack of breastfeeding and the morbidities of externalizing problems or ADHD were eliminated in this model. These trends preserved even after adjusting for maternal IQ in addition to the child’s IQ and other covariates (Model 3) (Table 2).

Linear regression analyses revealed that after adjusting for gender, age, residential area, and yearly family income, a lack of breastfeeding was associated with lower child’s IQ (unstandardized coefficient *B* = -4.17, 95% CIs = -6.07 to -2.28). This association slightly weakened but remained significantly after adjusting for maternal IQ (*B* = -3.82, 95% CIs = -5.23 to -1.68) and even after adjusting for the diagnosis of ADHD in addition to maternal IQ and other covariates (*B* = -3.32, 95% CIs = -5.10 to -1.55).

## Discussion

In this study, we found that a lack of breastfeeding was associated with increased morbidity of ADHD and internalizing and externalizing behavioral problems and low intelligence in childhood. Protective effect of breastfeeding on these behavioral problems might be partially mediated by the child’s IQ, whereas positive effect of breastfeeding on intelligence might be partially mediated by attention problem of children.

Existing research on the effect on breastfeeding on behavioral problems tends to focus on infancy and early childhood. Positive behavioral outcomes in breastfed infants include a greater degree of engagement and emotional regulation [22], fewer abnormal reflexes, signs of depression and withdrawal [23], and more alertness during social interactions [24]. In contrast, the results from a large, cluster-randomized trial did not find significant differences in behavioral outcomes at age 6 for those infants whose mothers were encouraged to breastfeed exclusively and for longer durations compared to infants whose mothers were not encouraged to do this [6]. In a pregnancy cohort study followed for 14 years, a shorter duration of breastfeeding was associated with increased morbidity of externalizing, internalizing, and overall behavioral problems, as measured by the CBCL [10]. The cited study controlled for several confounding variables, including family, social, economic, birth, and psychological factors in early life, but the study did not investigate the confounding effect of the mother’s or child’s IQ. Consistent with previous studies, we found a significant association between a lack of breastfeeding and the increased morbidities of externalizing, internalizing, and overall behavioral problems. The mother’s and child’s IQs explained some of the effect of breastfeeding, but not all. In particular, a lack of breastfeeding has sizable impact on internalizing or overall behavioral problems, independent of its effect on the child’s IQ.

ADHD is a common externalizing behavioral disorder that affects 8% to 12% of school-aged children [25]. ADHD is characterized by symptoms of inattention and/or hyperactivity/impulsivity. With an estimated heritability of approximately 75%, ADHD is generally regarded as having a genetic basis [26]. However, the remaining phenotypic variance (25%) in ADHD has been largely attributed to environmental factors, and several modifiable environmental risk factors have been suggested, such as tobacco smoke or alcohol exposure in pregnancy [27]. Previously, two studies examined the association between breastfeeding and ADHD. In one study, authors reported that the mean duration of breastfeeding was shorter in children with ADHD than that in controls (0.45 years vs. 0.55 years) [9]. In another study, authors found a significant association between ADHD and lack of breastfeeding at 3 months of age (OR = 3.08, 95% CI 1.46-6.50) [8]. However, neither study considered the confounding effect of the mother’s or child’s IQs.

Several possible bidirectional or interactive relationships could link breastfeeding with the presence of ADHD and the child’s IQ. One possibility is that lack of breastfeeding decreases intelligence [4, 7] and low child’s IQ increases the likelihood of ADHD [13]. Other possibility is that lack of breastfeeding increases the likelihood of ADHD [8, 9] and attention problem of children decreases the child’s IQ [14, 15]. Consistent with previous studies [8, 9], we found a significant association between lack of breastfeeding and ADHD, but this association was not statistically significant after controlling for the child’s IQ. A recent review of research on attention problems suggested that such research should control for IQ performance and, thereby, control for the potential influence of cognitive competence on ADHD symptoms [12]. We also found a significant association between lack of breastfeeding and low intelligence, in line with previous studies [4, 7]. This association weakened, but remained significant even after controlling for the presence of ADHD, suggesting sizable impact of breastfeeding on the child’s IQ, independent of its effect on the child’s attention problem.

This study has several limitations. First, due to the information on breastfeeding being based on the mothers’ recollection of the children, the respondents’ reports may be characterized by inaccuracies. Second, the small sample size of the morbidity group did not provide sufficient statistical power to detect modest differences. Third, we did not obtain information on the duration of breastfeeding, and we classified infant feeding only as ever being breastfed or never being breastfed. Failure to account for breastfeeding duration could lead to an underestimation of the true effect of breastfeeding on a child’s behavior. Finally, we did not obtain information on maternal education, home environment, maternal affective state, or mother-child attachment, which may have confounded the association between a lack of breastfeeding and child’s internalizing problems. Therefore, future prospective studies with detailed data regarding breastfeeding exposureand the number of additional confoundersare required to confirm our findings.

Despite these limitations, this is the first investigation of the effect of breastfeeding on behavioral and cognitive problems using an adequate control for maternal IQ and the child’s IQ or attention problem. Our findings suggest that there is a protective effect of breastfeeding on childhood behavioral outcomes with a partial mediation of this effect by the child’s IQ, and there is a positive effect of breastfeeding on childhood intelligence with a partial mediation of this effect by the child’s attention problem.

## Abbreviations

ADHD: Attention-deficit hyperactivity disorder
DISC-IV: Diagnostic interview schedule for children version IV
CBCL: Child behavior checklist
KEDI-WISC: Korean Educational Development Institute’s Wechsler Intelligence Scales for Children
K-WAIS: Korean Wechsler adult intelligence scale.
